# Impact of Age and Comorbid Conditions on Incidence Rates of COVID‐19‐Associated Hospitalizations, 2020–2021

**DOI:** 10.1111/irv.70016

**Published:** 2024-11-17

**Authors:** Lisa Saiman, Edward E. Walsh, Angela R. Branche, Angela Barrett, Luis Alba, Sonia Gollerkeri, Julia A. Schillinger, Matthew Phillips, Lyn Finelli

**Affiliations:** ^1^ Department of Pediatrics Columbia University Irving Medical Center New York New York USA; ^2^ Department of Infection Prevention & Control New York‐Presbyterian Hospital New York New York USA; ^3^ Department of Medicine, Division of Infectious Diseases University of Rochester Rochester New York USA; ^4^ Department of Medicine Rochester General Hospital Rochester New York USA; ^5^ Merck & Co., Inc Rahway New Jersey USA

**Keywords:** age and comorbid conditions, COVID‐19, incidence rate of hospitalization

## Abstract

**Background:**

COVID‐19‐associated hospitalization rates by age and comorbid conditions can more precisely assess risk for severe illness and target prevention and treatment strategies.

**Methods:**

We performed a retrospective study to estimate population‐based COVID‐19‐associated hospitalization among patients by age and selected comorbid conditions in three hospital systems in Rochester and New York City (NYC), NY. Incidence rate ratios (IRR) comparing incidence rates for patients with and without these comorbidities were determined.

**Results:**

From March 2020 to December 2021, 7779 patients were hospitalized with COVID‐19 of whom 43.8% had ≥3 comorbid conditions. Overall annual incidence ranged from 325.3 to 965.8 per 100,000 persons. Age group‐specific incidence was lowest in children 10–14 years (range 4.4–58.9) and highest in adults ≥85 years (range 2790.5–5889.6). Incidence rates for comorbid conditions generally increased with increasing age while IRR decreased with increasing age. Children in NYC 5–17 years with asthma or obesity had 3.4 and 53.3 times higher hospitalization rates, respectively, than children without these conditions. Adults in all age groups with obesity, diabetes, coronary artery disease, or congestive heart failure CHF had 1.6–4.7 times, 1.7–7.2 times, 2.0–10.1 times, or 1.7–20.2 times higher hospitalization rates, respectively, than those without these conditions. Adults ≥50 years with asthma had 1.5 to 1.8 times higher hospitalization rates than those without asthma.

**Conclusions:**

The burden of hospitalization with COVID‐19 was high, particularly among adults ≥85 years and adults with obesity, diabetes, CAD, or CHF. However, the impact of comorbidities was less in older adults. Population‐based incidence rates by age and comorbidities provide more precise estimates of the benefits of vaccines and antiviral medications.

## 1 Introduction

The COVID‐19 pandemic began in the United States in March 2020 resulting in 3,616,743 hospitalizations from May 2020 through April 2021 [1]. However, few studies have assessed the population‐based incidence of COVID‐19‐associated hospitalizations in patients of different age groups and comorbidities that confer a higher risk for severe COVID‐19 illness [2]. This study's objectives were to estimate [1] population‐based incidence of COVID‐19‐associated hospitalizations in predefined surveillance areas in New York State from March 2020 through December 2021, [2] incidence of hospitalization for different age groups, and [3] incidence rate ratios (IRR) in hospitalized patients in different age groups with and without selected comorbid conditions.

## 2 Methods

### 2.1 Study Design, Sites, and Eligible Patients

We conducted a retrospective review of COVID‐19‐associated hospitalizations from March 2020 through December 2021 at three hospital systems: NewYork‐Presbyterian Hospital, Columbia University Irving Medical Center (NYP‐CUIMC) in New York City (NYC) and the University of Rochester Medical Center, Strong Memorial Hospital (URMC) and Rochester Regional Health System, Rochester General Hospital (RGH) in Rochester, NY. The NYC surveillance area was defined as eight zip codes adjacent to or near the hospitals (10032, 10033, 10034, 10040, 10452, 10453, 10463, and 10471) in which we had previously estimated population‐based incidence of hospitalizations due to respiratory syncytial virus (RSV) infections [3]. The Rochester surveillance area was defined as patients residing in Monroe County for which URMC and RGH collectively held approximately 66% market share of internal medicine discharges. Market share was provided by hospital financial offices, and for the Rochester site verified by review of the Monroe County Statewide Planning and Research Cooperative System (SPARCS) data for COVID‐19 admissions.

Included patients were admitted for at least 24 h; resided in the pre‐defined surveillance areas; had one or more of the following: fever ≥37.8°C or feeling feverish, chills/rigors, cough, hoarseness, wheezing, shortness of breath, chest pain, sore throat, runny nose/nasal congestion, headache, loss of/altered sense of smell, loss of/altered sense of taste, malaise, myalgia/body aches, abdominal pain, nausea/vomiting or diarrhea; and had a positive reverse transcriptase‐polymerase chain reaction (RT‐PCR) test for SARS‐CoV‐2 within 14 days prior to admission or within 3 days after admission. Starting in mid‐March 2020, all patients were routinely tested on admission for SARS‐CoV‐2 by RT‐PCR performed on nasal swabs; tests changed throughout the study period due to availability of different assays. The study was approved by the institutional review boards of the three participating hospitals with a waiver of informed consent.

### 2.2 Data Collection

Demographic and clinical characteristics including preexisting comorbid conditions, healthcare resource utilization (HCRU), and outcome data, including all‐cause mortality, were extracted from the electronic medical record (EMR) using data queries and chart review. Comorbid conditions for NYC patients were only collected in 2021. HCRU included maximal respiratory support during hospitalization, ICU admission, obtained chest radiographs and/or chest CT scans, and use of selected medications. All‐cause readmissions within 1 year after discharge for surviving patients admitted in 2020 and within 6 months for those admitted in 2021 were assessed. Blood cultures obtained throughout hospitalization and respiratory cultures obtained during the first 7 days of hospitalization that grew pathogenic bacteria were collected. Patients' living situation at admission and discharge were collected to compare the level of care at admission versus discharge as previously described [4]. Admission living situation for NYC patients was only collected in 2021.

### 2.3 Data Analysis

For all analyses, data from the two Rochester sites were combined. Chi‐square and Wilcoxon tests compared categorical and continuous variables, respectively. Population‐based SARS‐CoV‐2 incidence rates of hospitalization per 100,000 population and 95% confidence intervals (95% CI) were determined for all ages collectively, 4 pediatric age groups (0–4, 5–9, 10–14, and 15–17 years of age), and 5 adult age groups (18–49, 50–64, 65–74, 75–84, and ≥85 years of age).

Population‐based incidence of hospitalization by age group was estimated by dividing the number of eligible patients by the adjusted 2020 US Census population estimate for the surveillance areas, adjusted by the hospitals' percent market share. For NYC, the incidence was calculated for each zip code using the market share of each zip code, and data were then aggregated for final incidence rates.

Population‐based incidence rates were estimated for hospitalized children <18 years of age with and without asthma and with and without obesity (only NYC children in 2021), and for hospitalized adults with and without asthma, obesity, diabetes, chronic obstructive pulmonary disease (COPD), congestive heart failure (CHF), and coronary artery disease (CAD). All patients were included in individual estimates and those with multiple comorbidities contributed to each individual comorbidity incidence estimate. The denominators for these calculations were determined for each age group using the 2020 US census data and for age group‐specific proportions of children and adults living with specific comorbidities which included the 2021 NYS Behavioral Risk Factor Surveillance System (BRFSS) data for asthma, obesity, diabetes, COPD, and CAD [5] and the National Health and Nutrition Examination Survey (NHANES) for CHF [6] (Table S1). Denominators were adjusted by market share. These data were then used to estimate IRRs with 95% CI for each comorbid condition for selected age groups.

The Wald method for normal distribution was used to estimate 95% CI when case counts were ≥5. Exact binomial calculations using the Clopper–Pearson methods were used when case counts were <5. Calculations were completed in Excel and Stata.

## 3 Results

### 3.1 Characteristics of Study Population

From March 11, 2020, to December 31, 2022, 7779 hospitalized patients (4496 from the Rochester sites and 3283 from the NYC site) were included for analysis (Table 1). Their median age ranged from 61.4 to 69.0 years. The percentage of patients 0–17 years increased from 1.4% in 2020 to 3.2% in 2021. The highest percentage of cases occurred in those 50–64 years (22.5%–28.9%, range in both sites for both years). Most patients in Rochester were white non‐Hispanic (49.7%–50.3%, range for both years) or Black/African American (31.1%–33.0%, range for both years), while most patients in NYC were Hispanic (60.8%–61.2%, range for both years).

**TABLE 1:** Characteristics of the study population.

	2020		2021	
Characteristics	Rochester, NY (*N* = 1622)	New York City (*N* = 1983)	Rochester, NY (*N* = 2874)	New York City (*N* = 1300)
Demographic characteristics				
Age, median (IQR), years	66 (54.5, 77)	69.0 (56.1, 79.6)	61.4 (46.3, 73.7)	67.0 (51.4–78.7)
Age groups, years, *n* (%)				
0–4	12 (0.7%)	13 (0.7%)	49 (1.7%)	18 (1.4%)
5–9	3 (0.2%)	3 (0.2%)	7 (0.2%)	4 (0.3%)
10–14	2 (0.1%)	3 (0.2%)	22 (0.8%)	7 (0.5%)
15–17	10 (0.6%)	4 (0.2%)	23 (0.8%)	3 (0.2%)
18–49	278 (17.1%)	327 (16.5%)	729 (25.4%)	275 (21.2%)
50–64	449 (27.7%)	477 (24.1%)	830 (28.9%)	293 (22.5%)
65–74	382 (23.6%)	465 (23.4%)	574 (20.0%)	264 (20.3%)
75–84	311 (19.2%)	403 (20.3%)	398 (13.8%)	259 (19.9%)
≥85	175 (10.8%)	288 (14.5%)	240 (8.4%)	177 (13.6%)
Male, *n* (%)	825 (50.9%)	1065 (53.7%)	1417 (49.3%)	638 (49.1%)
Race, *n* (%)				
White (non‐Hispanic)	815 (50.3%)	193 (9.7%)	1429 (49.7%)	121 (9.3%)
Black or African American (non‐Hispanic)	505 (31.1%)	189 (9.5%)	948 (33.0%)	170 (13.1%)
Hispanic	209 (12.9%)	1205 (60.8%)	364 (12.7%)	795 (61.2%)
Asian	39 (2.4%)	13 (0.7%)	50 (1.7%)	5 (0.4%)
Native Hawaiian or Pacific Islander	0 (0.0%)	8 (0.4%)	3 (0.1%)	1 (0.1%)
Native American/Alaska Native	3 (0.2%)	0 (0.0%)	5 (0.2%)	0 (0.0%)
Unknown/Declined	236 (14.6%)	242 (12.2%)	404 (14.1%)	130 (10.0%)
Other not described/more than one race	24 (1.5%)	133 (6.7%)	35 (1.2%)	78 (6.0%)
Underlying medical conditions, *n* (%)		(Not collected)		
Asthma	330 (20.0%)		596.6 (20.7%)	196 (15.1%)
Chronic obstructive pulmonary disease	222 (13.7%)		342 (11.9%)	84 (6.5%)
Congestive heart failure	374 (23.1%)		562 (19.6%)	139 (10.7%)
Coronary artery disease	450 (27.7%)		689 (24.0%)	186 (14.3%)
Obesity BMI ≥ 30	869 (53.6%)		1528 (53.2%)	444 (34.2%)
BMI ≥40	266 (16.3%)		474 (20.8%)	197 (15.2%)
Diabetes mellitus	694 (42.9%)		1135 (39.3%)	449 (34.5%)
Chronic kidney disease	489 (30.2%)		742 (25.8%)	198 (15.2%)
Dialysis	67 (4.1%)		128 (4.5%)	35 (2.7%)
Chronic liver disease	233 (14.4%)		405 (14.1%)	32 (2.5%)
HIV positive	28 (1.7%)		29 (1.1%)	27 (2.1%)
History of cancer	407 (25.1%)		677 (23.6%)	129 (9.9%)
Dementia	227 (14.0%)		220 (7.7%)	137 (10.5%)
Neuromuscular disorders	372 (22.9%)		677 (23.6%)	130 (10.0%)
Stroke	236 (14.5%)		349 (12.1%)	114 (8.8%)
Number of underlying medical conditions, *n* (%)		(Not collected)		
0	407 (25.1%)		407 (13.7%)	202 (15.5%)
1	277 (17.1%)		497 (17.3%)	228 (17.5%)
2	275 (17.0%)		514 (17.9%)	451 (34.7%)
3 or more	663 (40.9%)		1456 (50.7%)	419 (32.2%)
Baseline living situation, *n* (%)		(Not collected)		
Home independently	1232 (76.0%)		2393 (83.3%)	954 (73.4%)
In community with assistance of family or aide	90 (5.5%)		241 (8.4%)	291 (22.4%)
Assisted living	67 (4.1%)		67 (2.3%)	
Skilled nursing/rehabilitation facility	219 (13.5%)		95 (3.3%)	54 (4.2%)
Other	20 (1.2%)		64 (2.2%)	
Unknown	3 (0.1%)		7 (0.2%)	1 (0.1%)

Three or more comorbid conditions were present in 32.2%–50.7% (range in both sites for both years) of patients. Obesity (BMI ≥ 30) and diabetes mellitus were the most common comorbid conditions. Prior to hospitalization most patients lived independently, although a greater percentage of patients in NYC lived in the community with assistance from family or aides.

### 3.2 Population‐Based Incidence of COVID‐19‐Associated Hospitalization

**FIGURE 1:**
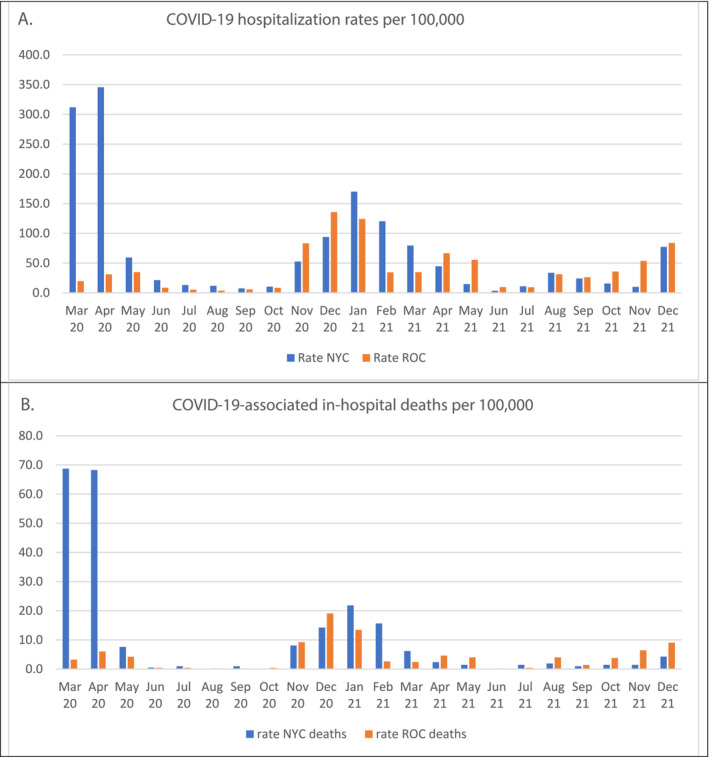
Monthly COVID‐19 hospitalizations per 100,000 persons (A) and COVID‐19 associated in‐hospital deaths per 100,000 persons (B) in New York City (NYC) and Rochester, NY (ROC) study sites, March 2020 to December 2021.

The monthly population‐based incidence rates and number of hospitalizations at both sites reflected the pandemic's onset in March 2020 and three distinct waves of COVID‐19 illness (Figure 1A and Figure S1A). Overall annual incidence per 100,000 was 325.3 and 576.4 in Rochester and 965.8 and 633.1 in NYC, in 2020 and 2021, respectively. During the first wave, the incidence rate and number of hospitalizations were higher in NYC compared to Rochester, while during the second wave (November 2020–May 2021) and start of the third wave (August–December 2021), rates were more similar.

In adults, the incidence rate of COVID‐19‐associated hospitalizations increased at both sites with age, while in children, incidence rates were generally highest in the 0‐ to 4‐year‐old age group at both sites in both years (Table 2 and Figure S2). For all age groups, incidence rates were greater in NYC in 2020 and increased in Rochester in 2021.

**TABLE 2:** Incidence of COVID‐19‐associated hospitalizations per 100,000 persons by age group, 2020–2021.

	Rochester, NY (2020)	New York City (2020)	Rochester, NY (2021)	New York City (2021)
Age groups (years)	Incidence rate (95% CI)		Incidence rate (95% CI)	
0–4	30.4 (13.2, 47.7)	101.8 (46.5, 157.2)	124.3 (89.5, 159.1)	141.0 (75.9, 206.1)
5–9	7.1 (0, 15.2)	24.9 (5.1, 72.6)	16.6 (4.3, 28.9)	33.2 (9.0, 84.9)
10–14	4.4 (0, 10.4)	25.2 (5.2, 73.8)	48.1 (28.0, 68.1)	58.9 (15.3, 102.5)
15–17	36.3 (13.8, 58.8)	56.5 (15.4, 144.6)	83.6 (49.4, 117.7)	42.4 (8.7, 123.8)
18–49	153.8 (135.7, 171.9)	343.6 (306.4, 380.8)	403.3 (374.0, 432.5)	289.0 (254.9, 323.1)
50–64	499.6 (453.4, 545.8)	1347.4 (1227.3, 1467.5)	923.5 (860.7, 986.3)	827.7 (733.3, 922.1)
65–74	854.7 (769.0, 940.4)	2732.7 (2487.7, 2977.7)	1284.3 (1179.2, 1389.4)	1551.5 (1365.8, 1737.2)
75–84	1412.3 (1255.3, 1569.2)	4448.1 (4023.6, 4872.7)	1807.3 (1629.8, 1984.9)	2858.8 (2515.7, 3202.0)
≥85	2790.5 (2377.0, 3203.9)	5889.6 (5229.7, 6549.4)	3826.9 (3342.8, 4311.1)	3619.4 (3095.9, 4142.9)
All	325.3 (309.5, 341.2)	965.8 (923.5, 1008.1)	576.4 (555.4, 597.5)	633.1 (598.8, 667.4)
≥18	487.6 (463.7, 511.5)	1112.4 (1063.4, 1161.3)	847.1 (815.5, 878.6)	719.6 (680.2, 759.1)
≥65	1234.9 (1152.8, 1317.1)	3733.1 (3522.0, 3944.3)	1727.0 (1630.2, 1824.3)	2260.5 (2095.0, 2426.1)

### 3.3 IRRs in Persons With and Without Comorbid Conditions

The estimated incidence of COVID‐19‐associated hospitalization in persons with and without selected comorbid conditions was used to calculate IRRs (Table 3). An estimated IRR > 1.0 is interpreted as an excess risk of hospitalization for persons with a specific condition when compared to those without the condition. While the incidence of hospitalization in persons with each of the selected comorbid conditions increased with increasing age, IRRs did not follow this pattern as they were generally higher in younger adults.

**TABLE 3:** Incidence rates of COVID‐19‐associated hospitalizations per 100,000 persons in patients with and without selected underlying medical conditions and incidence rate ratios.^a^ ^,^ ^b^

	Rochester, NY, 2020			Rochester, NY, 2021			New York City, 2021		
Age groups (years)	Incidence rate	Incidence rate	Incidence rate ratio (95% CI)	Incidence rate	Incidence rate	Incidence rate ratio (95% CI)	Incidence rate	Incidence rate	Incidence rate ratio (95% CI)
	**Asthma**	**No asthma**		**Asthma**	**No asthma**		**Asthma**	**No asthma**	
5–17	20.5	12.6	1.75 (0.5–6.2)	121.7	52.2	**3.1 (1.9–5.6)**	114.4	33.8	**3.4 (1.1, 10.1)**
18–49	182.1	146.7	1.2 (0.9, 1.6)	482.8	651.1	0.74 (0.06, 0.9)	423.2	266.4	**1.6 (1.2, 2.1)**
50–64	734.4	454.7	**1.6 (1.3, 2.0)**	1295.6	1317.6	0.98 (0.8, 1.15)	864.7	821.3	1.1 (0.8, 1.4)
≥65	2180.3	1227.5	**1.8 (1.5, 2.1)**	3075.5	2213.6	**1.5 (1.2, 6.0)**	2136.7	2279.5	0.9 (0.7, 1.2)
	**Obesity**	**No obesity**		**Obesity**	**No obesity**		**Obesity**	**No obesity**	
5–17	Unavailable^c^			Unavailable^c^			213.7	4.0	**53.3 (7.0, 407.7)**
18–49	310.1	65.7	**4.7 (3.6, 6.2)**	701.6	3.1	**2.3 (2.0, 2.7)**	599.8	194.5	**3.1 (2.4, 3.9)**
50–64	769.8	311.4	**2.5 (2.0, 3.0)**	1417.6	1.8	**1.6 (1.4, 1.8)**	1209.3	670.3	**1.8 (1.4, 2.8)**
≥65	11836.5	1095.8	**1.7 (1.5, 1.9)**	2669.5	0.9	1.02 (0.9, 1.2)	2170.1	2290.7	0.9 (0.8, 1.1)
	**Diabetes**	**No diabetes**		**Diabetes**	**No diabetes**		**Diabetes**	**No diabetes**	
18–49	838.2	115.6	**7.2 (5.6, 9.4)**	1686.8	447.4	**3.8 (3.2, 4.5)**	1073.7	251.1	**4.3 (3.1, 5.9)**
50–64	1332.6	329.6	**4.0 (3.3, 4.9)**	2448.5	941.8	**2.6 (2.3, 3.0)**	1500.7	667.8	**2.2 (1.8, 2.9)**
≥65	3061.1	880.4	**3.5 (3.0, 4.0)**	4439.0	2067.1	**2.1 (1.9, 2.4)**	3197.0	1859.2	**1.7 (1.5, 2.0)**
	**COPD**	**No COPD**		**COPD**	**No COPD**		**COPD**	**No COPD**	
18–49	108.8	155.4	0.7 (0.3, 1.5)	295.2	562.7	0.5 (0.3, 0.8)	55.3	293.5	0.2 (0.03, 1.3)
50–64	602.7	489.9	1.2 (0.9, 1.7)	1229.6	13.81.5	0.9 (0.7, 1.1)	727.6	834.7	0.9 (0.5, 1.4)
≥65	2095.24	1224.2	**1.7 (1.4, 2.0)**	2823.5	2947.4	0.96 (0.8, 1.1)	2449.8	2242.5	1.1 (0.8, 1.4)
	**CAD**	**No CAD**		**CAD**	**No CAD**		**CAD**	**No CAD**	
18–49	1443.1	144.8	**10.0 (6.2, 16.2)**	3848.1	379.3	**10.1 (7.6, 13.6)**	NA	285.8	NA
50–64	2806.7	410.7	**7.1 (5.6, 8.9)**	6000.9	736.1	**8.2 (6.9, 9.6)**	1633.40	777.3	**2.1 (1.5, 3.0)**
≥65	4993.5	904.9	**5.5 (4.8, 6.3)**	6633.4	1306.2	**5.1 (4.5, 6.7)**	4112.56	2015.1	**2.0 (1.7, 2.4)**
	**CHF** ^**d**^	**No CHF**		**CHF** ^**d**^	**No CHF**		**CHF**	**No CHF**	
20–39	1663.5	116.0	**14.3 (6.3, 32.5)**	6931.1	342.9	**20.2 (13.5, 30.3)**	1002.6	236.8	4.2 (1.0, 17.1)
40–59	3257.4	322.4	**10.1 (7.5, 13.5**	6138.9	648.7	**9.5 (7.7, 11.7)**	2557.1	551.3	**4.6 (2.9, 7.4)**
60–79	4300.4	696.9	**6.2 (5.3, 7.2)**	5630.0	1083.0	**5.2 (4.5, 6.0)**	3208.1	1511.8	**2.1 (1.6, 2.8)**
≥80	6731.1	1748.3	**3.9 (3.0, 4.9)**	10374.6	2272.8	**4.6 (3.8, 5.5)**	5232.6	3042.1	**1.7 (1.3, 2.3)**

^a^ CAD, coronary artery disease; CHF, congestive heart failure; COPD, chronic obstructive pulmonary disease.

^b^ Bolded cells indicate significant IRR whereby specific age groups with specific comorbidities are at increased risk of hospitalization when compared with those in the age groups without the specific comorbidities.

^c^ Unable to calculate the IRR for obesity in children 5–17 years in Rochester due to lack of standardized reporting by the school district.

^d^ Age groups available for CHF were different than age groups available for other comorbidities.

Children 5–17 years with asthma had 3.1 (Rochester in 2021) to 3.4 (NYC in 2021) times higher hospitalization rates than children without asthma. In NYC in 2021, children 5–17 years with obesity had a 53.3 times (95% CI 7.0, 407.7) higher hospitalization rate than children without obesity.

Adults in Rochester ≥50 years with asthma had 1.5 (in 2020) to 1.8 (in 2021) times higher hospitalization rates than adults without asthma. Adults with obesity 18–64 years had 1.6 to 4.7 times (both sites, both years) higher hospitalization rates than adults without obesity. Adults with diabetes in all age groups had 1.7 to 7.2 times (both sites, both years) higher hospitalization rates than adults without diabetes; the largest impact was noted among adults 18–49 years in Rochester in 2020. Adults with and without COPD had similar hospitalization rates.

Adults in all age groups with cardiac disease had the highest increased rates of hospitalization. Those with CAD had 2.0 to 10.1 times (both sites, both years) higher hospitalization rates than those without CAD; the greatest impact was among younger adults 18–49 years in Rochester in 2020 who had a 10.1 (95% CI 7.6, 13.6) times higher hospitalization rate. Those with CHF had 1.7 to 20.2 times (both sites, both years) higher hospitalization rates than those without CHF; the greatest impact was among younger adults 20–39 years in Rochester in 2021 who had a 20.2 (95% CI 13.5, 30.3) times higher hospitalization rate.

### 3.4 Mortality and Healthcare Resource Utilization

The in‐hospital all‐cause mortality in NYC was 18.2% in 2020 which declined to 10.1% in 2021 (*p* < 0.001) and in Rochester was 12.6% in 2020 and 9.5% in 2021 (*p* = 0.002) (Table 4). Death rates and number of deaths occurred in parallel with incidence rates and number of hospitalizations (Figure 1B and Figure S1B). In‐hospital mortality in adults increased with increasing age combining both sites for both years: 2.4% for 18–49 years, 6.3% for 50–59 years, 11.1% for 60–69 years, 19.9% for 70–84 years, and 26.0% for those ≥85 years. The population‐based mortality incidence per 100,000 during the 2‐year period combining both sites, was 13.7, 126.9, and 800.6 for those 18–49, 50–64, and ≥65 years, respectively. Thus, those 65 years of age and older were 58.4 times more likely to die than those 18–49 years of age.

**TABLE 4:** Healthcare resource utilization and outcomes in patients with COVID‐19‐associated hospitalizations, 2020–2021.^a^

	Rochester, NY, 2020 (*N* = 1622)	New York City, 2020 (*N* = 1983)	Rochester, NY, 2021 (*N* = 2874)	New York City, 2021 (*N* = 1300)
In‐hospital mortality, *n* (%)	205 (12.6%)	361 (18.2%)	274 (9.5%)	131 (10.1%)
Hospital length of stay, days (median, IQR)	7 (3, 15)	7 (4–12)	6 (3, 12)	7 (4–11)
Intensive care unit admission	466 (28.7%)	386 (19.5%)	694 (24.2%)	164 (12.6%)
Intensive care unit length of stay, days, (median, IQR)	7 (3, 14)	10 (3, 26)	7 (2, 17)	10 (3, 26)
Respiratory support, *n* (%)				
High flow O_2_	118 (7.3%)	5 (0.3%)	248 (8.6%)	4 (0.3%)
CPAP/Bi‐PAP	48 (3.0%)	30 (1.5%)	80 (2.8%)	49 (3.8%)
Mechanical ventilation	242 (14.9%)	306 (15.4%)	337 (11.7%)	102 (7.8%)
ECMO	8 (0.5%)	0 (0.0%)	19 (0.7%)	0 (0.0%)
Treatments prescribed within first 7 days of admission, *n* (%)				
Antibiotics	737 (45.4%)	1312 (66.2%)	971 (33.8%)	492 (37.8%)
Steroids	712 (43.9%)	348 (17.5%)	1554 (54.1%)	82 (6.3%)
Vasopressors	75 (4.6%)	330 (16.6%)	136 (4.7%)	83 (6.4%)
Remdesivir	514 (31.7%)	191 (9.6%)	948 (33.0%)	508 (39.1%)
Convalescent plasma	38 (2.3%)	0 (0%)	6 (0.2%)	NA (study only)
Monoclonal antibody	2 (0.1%)	0 (0%)	19 (0.7%)	
Microbiology, *n* (%)				
Positive blood culture during hospitalization	113 (7.0%)	153 (7.7%)	244 (8.5%)	54 (4.2%)
Positive respiratory culture during first 7 days of hospitalization	125 (7.7%)	31 (1.6%)	230 (8.0%)	30 (2.3%)
Radiographic studies performed during first 3 days of hospitalization, *n* (%)				
Chest radiograph	1399 (86.3%)	(Not collected)	2279 (79.3%)	1218 (93.7%)
CT scan	224 (13.8%)		497 (17. 3%)	188 (14.5%)
Survived to discharge	1417	1622	2600	1169
Discharge living situation in survivors				
Home independently	603 (42.6%)	840 (51.8%)	1407 (54.1%)	641 (54.8%)
In community with assistance of family or aide	519 (36.6%)	272 (16.8%)	775 (29.8%)	300 (25.7%)
Skilled nursing/rehabilitation facility/hospice	363 (25.6%)	461 (28.4%)	329 (12.7%)	204 (17.5%)
Psychiatric hospital/other	24 (1.7%)	30 (1.8%)	72 (2.8%)	3 (0.3%)
Higher level of care at discharge compared with level of care at admission	582 (34.1%)	(Not collected)	908 (34.9%)	296 (25.3%)
All cause readmission in survivors^b^	249 (17.6%)	463 (28.5%)	558 (21.5%)	268 (22.9%)

^a^ BiPAP, bilevel PAP; CPAP, continuous positive airway pressure; ECMO, extracorporeal membrane oxygenation; IQR, interquartile range; NA, not available; O_2_, oxygen.

^b^ Within 1 year of discharge for 2020 and within 6 months of discharge for 2021.

The median hospital length of stay was 6 (Rochester 2021) to 7 days (Rochester 2020 and NYC both years) (Table 4). In Rochester, ICU admissions were higher in both years. In NYC, high flow oxygen and extracorporeal membrane oxygenation were rarely used. The overall use of antibiotics at both sites during both years was common (33.8%–66.2%). Steroids were more often administered in Rochester (43.9%–54.1%, both years) than in NYC (6.3%–17.5%, both years). Remdesivir was administered to 9.6% of NYC patients in 2020, which increased to 39.1% in 2021, which was similar to remdesivir use in Rochester in both years (31.7% in 2020 and 33.0% in 2021). This may, in part, have been due to participation in a remdesivir treatment trial in Rochester.

During hospitalization at both sites for both years, 4.2%–8.5% of patients had a positive blood culture and during the first 7 days of hospitalization 1.6%–8.0% had a positive respiratory tract culture. Most patients (79.3%–93.7%) had chest radiographs obtained while CT scans were less frequent (13.8%–17.3%).

Among those who survived until discharge, 25.3% (NYC in 2021) to 34.9% (Rochester in 2021) were discharged to a higher level of care compared to their level of care at admission (Table 4). Combining data from both sites, all‐cause readmissions during the subsequent 12 months (year 2020 admissions) or 6 months (year 2021 admissions) occurred in 23.4% and 21.9% of survivors, respectively.

## 4 Discussion

Population‐based incidence studies allow comparisons of risk among patients in different age groups and with different comorbid conditions, and from different institutions and locales. Thus, incidence rates and IRRs derived from this study provide further insights into the groups at highest risk for COVID‐19‐associated hospitalization during the first 2 years of the COVID‐19 pandemic and enhance understanding of the patients who may have the greatest benefits from vaccines and antiviral medications.

Hospitalization rates per 100,000 population by age group estimated in this study were somewhat lower than the COVID‐Net age‐specific hospitalization rates reported from May 2020 to April 2021 [7, 8]. This difference could reflect our study's inclusion of the relatively low incidence months from May to December 2021. At the onset of the COVID‐19 pandemic, the overall population‐based incidence of hospitalizations in NYC was strikingly higher compared with Rochester. Rochester did not experience the early spring pandemic surge despite being only 334 miles from NYC. This observation is reminiscent of the epidemiology of the 1918 influenza pandemic whereby a herald wave of excess deaths, limited to NYC and thought to be influenza, occurred from February to April 1918, but the recognized pandemic onset in the United States occurred in the autumn of 1918 [9]. Studies of the onsets of both the 1918 and 2009 influenza A H1N1 pandemics noted surprisingly local and focal transmission [10]. As the COVID‐19 pandemic progressed, implementation of non‐pharmaceutical interventions likely resulted in more similar incidence rates in Rochester and NYC during the fall and winter of 2020. The development of increasing population‐based immunity due to high rates of infection in NYC during the first wave may have also impacted the incidence in NYC [11, 12].

As others have shown, the incidence rate of COVID‐19 hospitalizations in adults increased with age [13]. In this study, we observed an overall hospitalization prevalence of 1.2%–5.3% in adults ≥85 years. We also observed that incidence rates in older adults were much higher in NYC in 2020, which we speculate may have been due multigenerational households, crowded urban living situations, and a higher use of public transportation. Furthermore, increasing age has consistently been shown to be associated with increased mortality [8, 13]. We calculated that compared to those age 18–49 years, those ≥65 years were 58 times more likely to die during their COVID‐19‐associated hospitalization.

In addition to studying the frequency of comorbid conditions, one of the unique aspects of this study was assessing the impact of these conditions by estimating incidence rates and IRRs among those with and without the studied comorbid conditions. We found that the most common comorbid condition was obesity (49%). In both adults and children, obesity is a well‐described risk factor for severe outcomes from both COVID‐19 [14, 15] and the multisystem inflammatory syndrome in children [16]. While the strength of our findings are limited by the small pediatric sample size, strikingly, children in NYC 5–17 years with obesity had a 53.3 times higher hospitalization rate than children without obesity. In recent years, NYC public schools have focused on reducing obesity in school‐aged children to ameliorate the adverse health impacts of obesity throughout the lifespan [17, 18].

We found an interesting pattern for IRRs; except for asthma, higher IRRs were estimated for younger adults 18–49 years than for older adults. This trend was particularly pronounced for younger adults in Rochester with CAD and CHF as younger adults with selected conditions had a higher relative risk of hospitalization with COVID‐19 than older adults with the same conditions. These findings suggest that for older adults, age itself, presumably associated with frailty and immunosenescence, has a larger impact than comorbidities on hospitalization risk. We previously found similar trends in IRRs for adults with diabetes and CHF experiencing RSV‐associated hospitalizations [2]. Our data were also consistent with findings for influenza as IRRs for influenza‐associated hospitalization in younger adults with CHF and CAD were significantly higher than IRRs of older adults [19]. From March to June 2020, others similarly calculated rate ratios for COVID‐19‐associated hospitalizations using BRFSS estimates, and found higher adjusted hospitalization rate ratios for adults with obesity, chronic kidney disease, diabetes, hypertension, and asthma than adults without these conditions. In contrast to our findings, rate ratios adjusted for age, sex, race, and ethnicity were higher in the oldest adults ≥65 years of age compared with younger adults [20]. Different methodologies, study periods, and catchment areas likely contributed to the different findings.

The patterns observed in mortality and healthcare utilization between the two sites and from 2020 to 2021 may reflect differences in resources and potentially locally promulgated care patterns. For example, mortality rates associated with COVID‐19 were highest in NYC during 2020. Others have suggested that lower mortality rates over time were due, in part, to increased clinical experience caring for patients including the use of mechanical ventilation, prone positioning, steroids, and remdesivir [21]. Although the hospital length of stay was similar at both sites in both years, the percentage of patients admitted to the ICU was higher in Rochester and the median length of ICU hospitalization was somewhat higher in NYC. There is not a ready explanation for these observations, but perhaps different levels of care were provided in non‐ICU units in NYC or there may have been a lower threshold for ICU admission in Rochester. A less commonly assessed metric for healthcare utilization is the need for higher levels of care at hospital discharge when compared to hospital admission. While 73%–83% of patients were living at home independently prior to admission, among survivors, only 43%–55% were discharged to live at home independently and 25%–35% required an increased level of care. These findings are similar to our findings of the impact of RSV‐associated hospitalization [4].

The major strength of our study is our ability to estimate the population‐based risk of COVID‐19 hospitalization by underlying medical condition. To our knowledge, this is the first study to provide such estimates, during the first 2 years of the pandemic. We are confident about our case ascertainment as universal testing practices for COVID‐19 were in effect in all three hospital systems and we had prior experience characterizing all acute respiratory illnesses resulting in RSV‐associated hospitalizations [3]. Previously published studies relied on stimulated passive surveillance and may not have ascertained all COVID‐19 cases [22].

However, our study had limitations. It was conducted in NYS in three hospital systems and thus, the finding may not be generalizable. While we assessed patients for symptoms consistent with COVID‐19, it is possible that some patients had persistently positive SARS‐CoV‐2 RT‐PCR tests and were admitted for other reasons. We did not assess the impact of different SARS‐CoV‐2 variants. Comorbid conditions were extracted from the EMR and not otherwise confirmed. The calculations of incidence rates and IRR for different comorbidities did not account for the combined impact of more than one comorbidity. The estimated prevalence of underlying conditions was derived from the BRFSS which is limited to community dwelling adults; as 3.3%–13.5% of our patients resided in skilled nursing or rehabilitation facilities, the IRR could be overestimated. We were unable to assess the impact of socioeconomic status (SES) due to the limitations of available data, understanding the impact of SES would further our understanding of the contribution of various comorbidities, most notably obesity. Data for underlying conditions were not available for both sites for both years. The sample size for calculating the IRR for certain underlying conditions, for example, children with obesity, was small. HCRU varied, in part because sites did not share standardized treatment protocols. Lack of access to skilled nursing facilities could have delayed discharge, increased length of hospitalization, and impacted discharge disposition.

### 4.1 Summary and Conclusions

Population‐based COVID‐19 hospitalization incidence rates and IRRs provide insights into patient populations that could have the greatest benefits from vaccines and antiviral medications. Our findings could prove helpful for future comparisons as COVID‐19 becomes endemic. Furthermore, if vaccine uptake for COVID‐19 declines over time, findings can guide the public health community to focus vaccination efforts on those at highest risk.

## Author Contributions

**Lisa Saiman:** writing – review and editing, methodology, validation, conceptualization, investigation, funding acquisition, writing – original draft, supervision. **Edward E. Walsh:** conceptualization, investigation, funding acquisition, writing – original draft, methodology, validation, writing – review and editing, supervision. **Angela R. Branche:** conceptualization, investigation, funding acquisition, writing – original draft, methodology, validation, writing – review and editing, supervision. **Angela Barrett:** data curation, project administration, writing – review and editing. **Luis Alba:** writing – review and editing, formal analysis, data curation. **Sonia Gollerkeri:** formal analysis, data curation, writing – review and editing, validation, methodology. **Julia A. Schillinger:** writing – review and editing, methodology. **Matthew Phillips:** writing – review and editing, project administration. **Lyn Finelli:** conceptualization, methodology, investigation, funding acquisition, writing – original draft, validation, writing –review and editing.

## Conflicts of Interest

Lisa Saiman, Edward Walsh, and Angela Branche received grant support from Merck Sharp & Dohme LLC, a subsidiary of Merck & Co. Inc., Rahway, NJ, USA. Matthew Phillips, Julia Schillinger, and Lyn Finelli are employees of Merck Sharp & Dohme LLC, a subsidiary of Merck & Co. Inc., Rahway, NJ, USA who may own stock and/or hold stock options in Merck & Co. Inc., Rahway, New Jersey, USA.

## Supporting information


**Table S1.** Data Sources Used to Calculate Incidence Rates and Incidence Rate Ratios for Selected Comorbid Conditions.
**Figure S1.** Number of monthly COVID‐19 hospitalizations (A) and number of COVID‐19 associated in‐hospital deaths (B) in New York City (NYC) and Rochester, NY (ROC) study sites, March 2020 to December 2021.
**Figure S2.** Incidence rates for COVID‐19 hospitalizations by age groups in New York City (NYC) study site (A) and Rochester (ROC) study sites (B), March 2020 through December 2021.

## Data Availability

Data in the manuscript could be made available pending institutional review board approval.
