# Supplementary material for: Impact of Age and Comorbid Conditions on Incidence Rates of COVID‐19‐Associated Hospitalizations, 2020–2021
Source: Influenza Other Respir Viruses. 2024 Nov 17;18(11):e70016. doi: 10.1111/irv.70016 (PMC11569932; doi:10.1111/irv.70016)
Supplement: Supplementary file 1 — Table S1. Data Sources Used to Calculate Incidence Rates and Incidence Rate Ratios for Selected Comorbid Conditions. Figure S1. Number of monthly COVID‐19 hospitalizations (A) and number of COVID‐19 associated in‐hospital deaths (B) in New York City (NYC) and Rochester, NY (ROC) study sites, March 2020 to December 2021. Figure S2. Incidence rates for COVID‐19 hospitalizations by age groups in New York City (NYC) study site (A) and Rochester (ROC) study sites (B), March 2020 through December 2021. [file IRV-18-e70016-s001.docx]

**Supplemental Material**

**eTable 1: Data Sources Used to Calculate Incidence Rates and Incidence Rate Ratios for Selected Comorbid Conditions**

**eFigure 1.** Number of monthly COVID-19 hospitalizations and number of COVID-19 associated in-hospital deaths in New York City (NYC) and Rochester, NY (ROC) study sites, March 2020 to December 2021.

**eFigure 2.** Incidence rates for COVID-19 hospitalizations by age groups in New York City (NYC) study site and Rochester (ROC) study sites, March 2020 through December 2021.

**eTable 1: Data Sources Used to Calculate Incidence Rates and Incidence Rate Ratios for Selected Comorbid Conditions**

| **Comorbid Condition** | **Age Strata**  **(Years)** | **New York City**  **Data Source** | **Rochester**  **Data Source** | **Definition** |
| --- | --- | --- | --- | --- |
| Asthma | 5-17  18-49  50-64  ≥65 | 5-17 years - NYC DOHMH Division of School Health School Year 2018-2019, hospital catchment area^1^  >18 years - NYC DOHMH CHS, 2019-2020, city-wide^2^ | Rochester City School District (Monroe County)  >18 years - NY State BRFSS, 2021 | Ever asthma |
| Obesity >30 | 5-17  18-49  50-64  ≥65 | 5-17 years - NYC DOHMH Division of School Health  School Year 2018-2019, hospital catchment area^1^  >18 years - NYC DOHMH CHS, 2019-2020, city-wide^2^ | Monroe County school district  >18 years - NY State BRFSS, 2021 | Children: parameters measured in school  Adults: self-reported height and weight and BMI calculated |
| Diabetes | 18-49  50-64  ≥65 | >18 years - NYC DOHMH CHS, 2019-2020, city-wide^2^ | >18 years - NY State BRFSS^3^ | Ever diabetes |
| Chronic Obstructive Pulmonary Disease (COPD) | 18-49  50-64  ≥65 | NYC DOHMH CHS, 2019-2020, city-wide^2^ | NY State 2021 BRFSS | Ever COPD |
| Coronary Artery Disease (CAD) | 18-49  50-64  ≥65 | NY State BRFSS 2021 - NYC subset^3^ | NY State 2021 BRFSS^3^ | Ever CAD |
| Congestive Heart Failure (CHF) | 20-39  40-59  60-79  ≥80 | National data NHANES^4^ | National data NHANES^4^ | Ever CHF |

**Abbreviations in table**: - NYC DOHMH – New York City Department of Health and Mental Hygiene, CHS – Community Health Survey, BRFSS - Behavioral Risk Factor Surveillance System, NHANES – National Health and Nutrition Examination Survey

^1^Data source is a composite of school-based measures of asthma prevalence, and of heights and weights of students attending public schools in an aggregate of ZIP codes that comprise the hospital catchment area: 10032, 10033, 10034, 10040, 10452, 10453, 10463, and 10471

^2^Data source is the New York City (NYC) Community Health Survey (CHS) 2019-2020. The CHS includes adults with landlines as well as adults who can be reached only by cellphone. CHS 2019-2020 data are weighted to the adult residential population of NYC per the American Community Survey, 2019.

^3^NYS Behavioral Risk Factor Surveillance Survey (BRFSS)^5^

^4^Heart disease and stroke statistics—2020 update: A report From the American Heart Association^6^

**eFigure 1.** Number of monthly COVID-19 hospitalizations **(A)** and number of COVID-19 associated in-hospital deaths **(B)** in New York City (NYC) and Rochester, NY (ROC) study sites, March 2020 to December 2021.

**eFigure 2.** Incidence rates for COVID-19 hospitalizations by age groups in New York City (NYC) study site **(A)** and Rochester (ROC) study sites **(B)**, March 2020 through December 2021.
